# Supplementary material for: Global ubiquitinome profiling identifies NEDD4 as a regulator of Profilin 1 and actin remodelling in neural crest cells
Source: Nat Commun. 2022 Apr 19;13:2018. doi: 10.1038/s41467-022-29660-3 (PMC9018756; doi:10.1038/s41467-022-29660-3)
Supplement: Supplementary file 1 — Supplementary information [file 41467_2022_29660_MOESM1_ESM.pdf]

Supplementary Information

Global ubiquitinome profiling identifies NEDD4 as a regulator of Profilin 1 and actin remodelling in neural crest cells

Iman Lohraseb, Peter McCarthy, Genevieve Secker, Ceilidh Marchant, Jianmin Wu, Naveid Ali, Sharad Kumar, Roger J Daly, Natasha Harvey, Hiroshi Kawabe, Oded Kleifeld, Sophie Wiszniak & Quenten Schwarz[#].

#. Corresponding author. Email: quenten.schwarz@unisa.edu.au

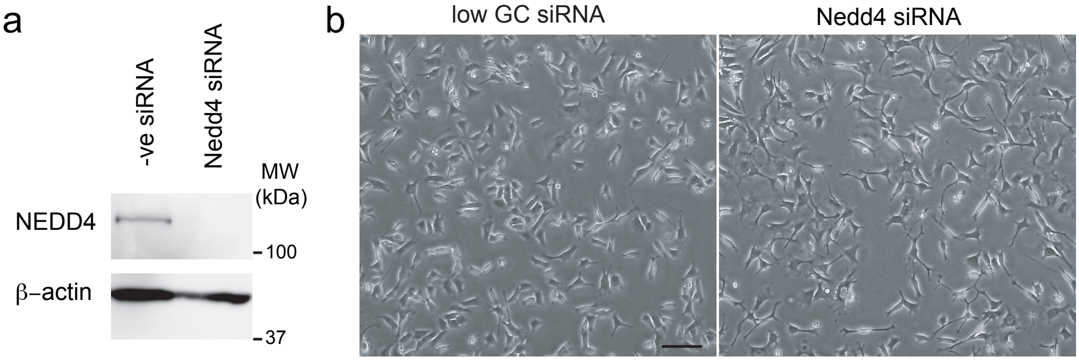

Supplementary Figure 1. Nedd4 siRNA knockdown leads to cell morphology changes. (a) Nedd4 siRNAs consistently lead to near complete knockdown of NEDD4 protein expression within 48hr compared to low GC control siRNAs. (b) Following Nedd4 knockdown, NCU10K NCCs undergo a significant morphological change, characterized by the extension of processes. Scale bar = 100μm. Blots and images are representative of 3 separate experiments. Source data are provided as a Source Data file.

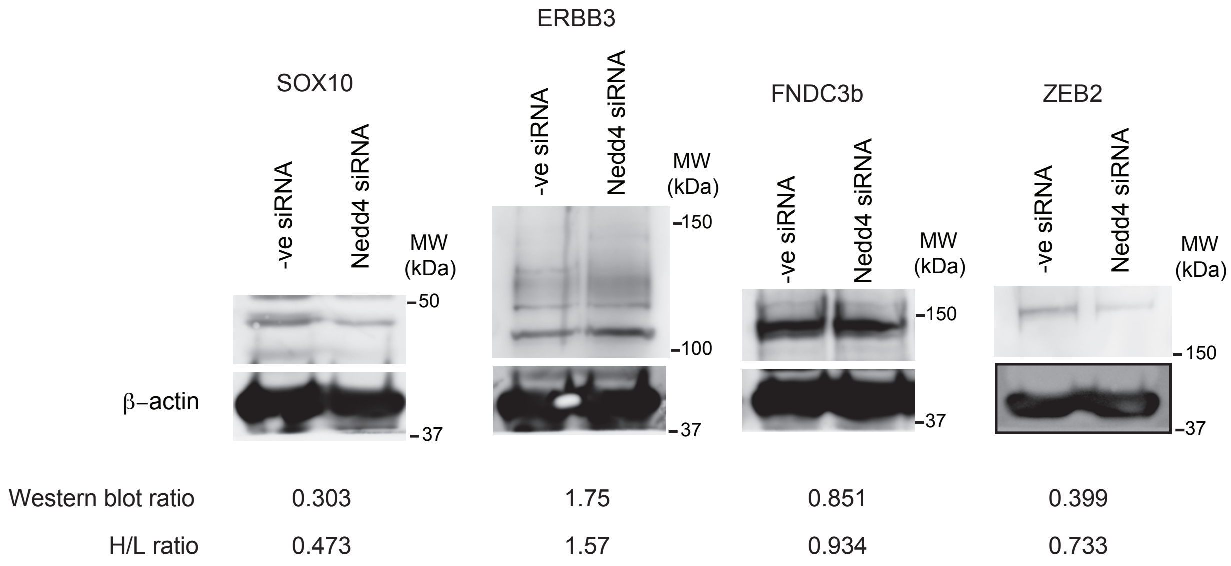

Supplementary Figure 2. Validation of relative protein abundance for four randomly selected proteins identified by proteomics analysis. Lysates from NCU10K cells following 48hrs Nedd4 or low GC control siRNA knockdown (-ve siRNA) immunoblotted with the specified antibodies to SOX10, ERBB3, FNDC3b and ZEB2. The H/L ratio value comes from the unenriched total protein quantitation alongside densitometry of western blots normalized to β-actin controls. β-actin load control for ZEB2 was obtained from samples derived from the same experiment with gels and blots processed in parallel. Blots are representative of 3 separate experiments. Uncropped images are provided as a Source Data file.

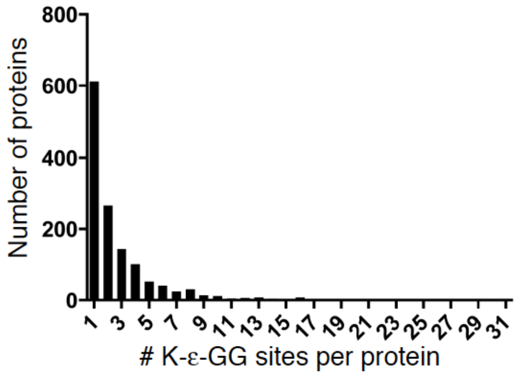

Supplementary Figure 3. Distribution of sites per protein observed in the ubiquitin remnant enrichment data. The distribution of proteins by number of identified ubiquitination sites per protein.

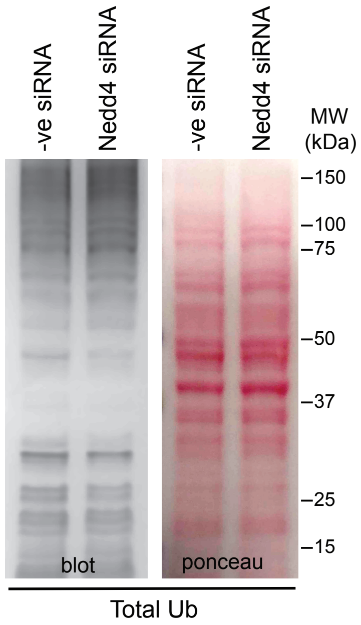

Supplementary Figure 4. Western blot for total ubiquitin levels in NCU10K cells treated with either low GC control (-ve siRNA) or Nedd4 siRNA. Lysates from NCU10K cells following 48hrs Nedd4 or low GC control siRNA knockdown immunoblotted for total ubiquitin (free and conjugated). Note that equal amounts of lysate loaded as indicated in Ponceau S stained membrane. Blots and images are representative of 3 separate experiments. Uncropped images are provided as a Source Data file.

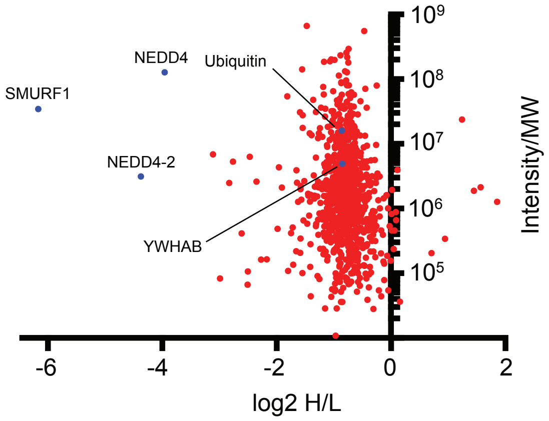

Supplementary Figure 5. Quantitative proteomic profiling of Nedd4 interactors. Frequency distribution of log2 H/L peptides enriched with NEDD4 co-immunoprecipitation. Peptides in blue are established interactors of NEDD4.

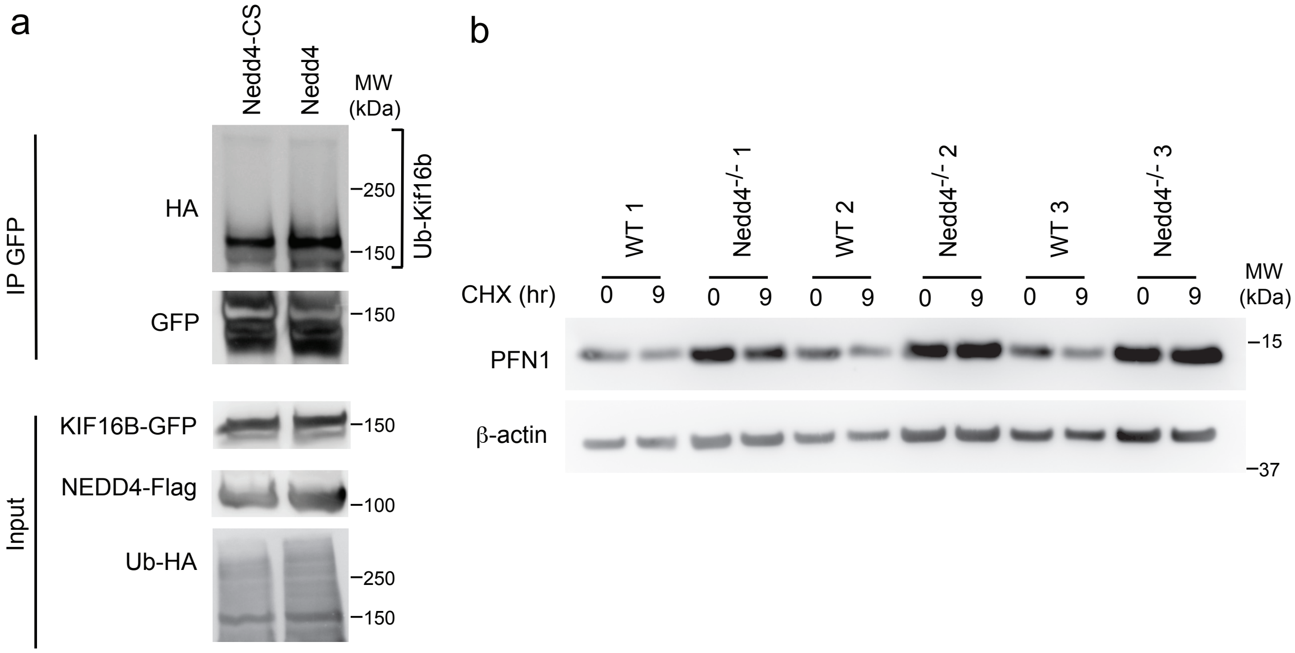

Supplementary Figure 6. Validation of Nedd4 substrates. (a) In vivo ubiquitination assay with 293T cells transfected with HA-Ub, Kif16b-GFP and Flag-Nedd4 or Flag-Nedd4-CS. Protein lysates were immunoprecipitated with GFP-trap beads. Ubiquitinated Kif16b was probed with anti-HA antibody. Uncropped images are provided as a Source Data file. Blots are representative of 3 separate experiments. (b) Cycloheximide chase analysis of Pfn1 degradation in WT and Nedd4$^{-/-}$ NCCs run on the same gel for quantitation in Figure 4F. Blots are representative of 3 separate experiments. Uncropped images are provided as a Source Data file.

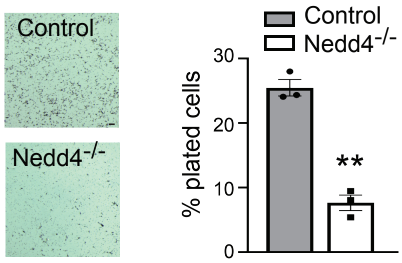

Supplementary Figure 7. Nedd4 regulates NCC migration. Representative images of Transwell migration assays with WT and Nedd4$^{-/-}$ NCCs. Scale bar = 100μm. Quantitation of the percentage of cells that migrated through the Transwell is shown for all CRISPR cell lines, n=3 independent experiments. Data are presented as mean +/-SEM. **, P=0.0005 as determined by type one Student's t test. Source data are provided as a Source Data file.

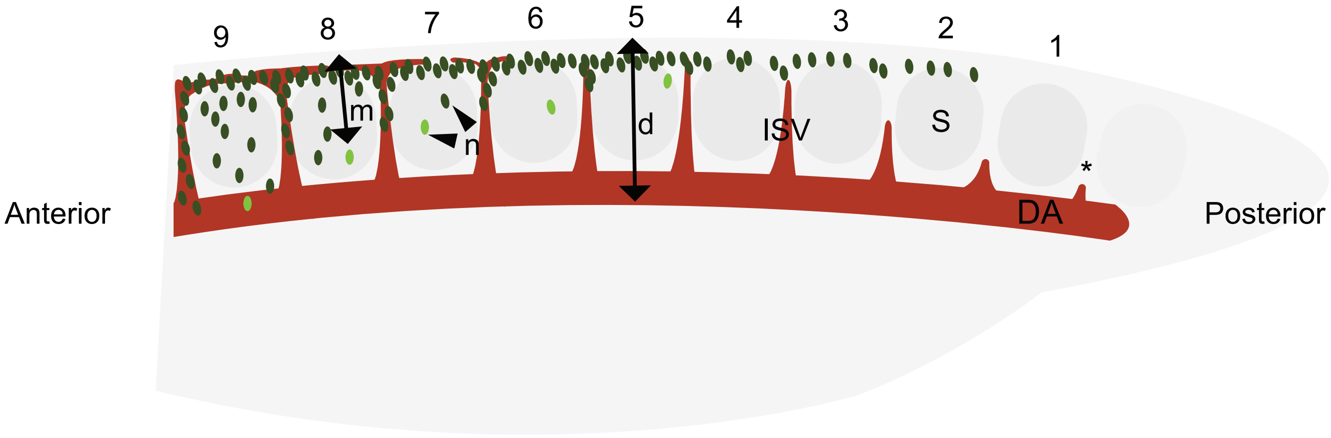

m = distance from dorsal limit of the embryo to the furthest (pioneer) NCCs (light green) within each somite
n = number of cells migrating in each somite
d = distance from dorsal limit of the embryo to the ventral limit of the dorsal aorta

Supplementary Figure 8. Schematic of trunk NCC migration and quantitation parameters. At E9.5 trunk NCCs (green) delaminate from the dorsal neural tube and migrate within the somites (S) toward the dorsal aorta (DA). The migration distance of pioneering NCCs (light green) within each somite was measured from the dorsal limit of the embryo (m). To normalize migration across embryos and within somites the distance between the dorsal limit of the embryo and ventral limit of the dorsal aorta was also measured. The total number of NCCs within each somite was also counted (n). Normalisation of any developmental delay across genotypes was performed by counting somites from the presence of the first intersomitic vessel (ISV) sprouting from the dorsal aorta (asterics).

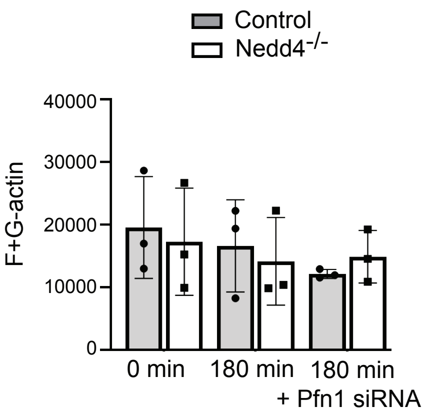

Supplementary Figure 9. Total actin is conserved in the absence of Nedd4. Quantitation of the F+G actin levels from all CRISPR cell lines after CytoD washout. This data matches the F/G actin ratios in Figure 7F, n=3 independent experiments. Data are presented as mean +/-SEM. Source data are provided as a Source Data file.
