# Supplementary material for: Global ubiquitinome profiling identifies NEDD4 as a regulator of Profilin 1 and actin remodelling in neural crest cells
Source: Nat Commun. 2022 Apr 19;13:2018. doi: 10.1038/s41467-022-29660-3 (PMC9018756; doi:10.1038/s41467-022-29660-3)
Supplement: Supplementary file 3 — Description of Additional Supplementary Files [file 41467_2022_29660_MOESM3_ESM.docx]

**Supplementary data legends**

File Name: Supplementary Data 1.

Description: Full list of identified and quantified proteins with supporting data. The complete list of protein groups identified and quantified in the pre-enrichment fractions from control and Nedd4 siRNA treated SILAC NCCs. Perseus was used to determine P values (Student’s t test) with Benjamini and Hochberg FDR applied to correct for multiple hypotheses testing (Significance B).

File Name: Supplementary Data 2.

Description: Full list of gene expression with supporting data. The complete list of genes analysed by microarray from 3 control and 3 Nedd4 siRNA treated NCCs. Raw P values were determined by two-sided ANOVA with adjustments made by Benjamini and Hochberg multiple comparisons (stepup P).

File Name: Supplementary Data 3.

Description: Molecular pathways enriched among differentially expressed genes. The complete list of molecular pathways identified from DAVID. P values were determined using Fisher’s Exact Test with adjustments using Bonferroni, Benjamini and FDR methods.

File Name: Supplementary Data 4.

Description: Molecular pathways enriched among differentially expressed proteins. The complete list of molecular pathways identified from DAVID. P values were determined using Fisher’s Exact Test with adjustments using Bonferroni, Benjamini and FDR methods.

File Name: Supplementary Data 5.

Description: Molecular pathways enriched among NEDD4 ubiquitinated targets. The complete list of molecular pathways identified from DAVID. P values were determined using Fisher’s Exact Test with adjustments using Bonferroni, Benjamini and FDR methods.

File Name: Supplementary Data 6.

Description: Full list of identified and quantified ubiquitin remnant peptides with supporting data. The complete list of identified and quantified ubiquitin remnant-enriched peptides from control and Nedd4 siRNA treated SILAC NCCs.

File Name: Supplementary Data 7.

Description: Protein interactions with ubiquitinated proteins determined with MS/MS analyses and PINA. This table shows which of the proteins identified as affected in the ubiquitin remnant screen also occur co-immunoprecipitate with Nedd4 or have known interactions as determined with PINA. A breakdown of how many interacting proteins are known vs novel is provided.

File Name: Supplementary Data 8.

Description: MS/MS analyses from Nedd4 immunoprecipitation analysis. This table shows which of the proteins identified as affected in the ubiquitin remnant screen also co-immunoprecipitate with Nedd4.
