# Supplementary material for: Global ubiquitinome profiling identifies NEDD4 as a regulator of Profilin 1 and actin remodelling in neural crest cells
Source: Nat Commun. 2022 Apr 19;13:2018. doi: 10.1038/s41467-022-29660-3 (PMC9018756; doi:10.1038/s41467-022-29660-3)
Supplement: Supplementary file 12 — Reporting Summary [file 41467_2022_29660_MOESM12_ESM.pdf]

nature portfolio

Corresponding author(s): Quenten Schwarz

Last updated by author(s): Mar 1, 2022

Reporting Summary

Nature Portfolio wishes to improve the reproducibility of the work that we publish. This form provides structure for consistency and transparency in reporting. For further information on Nature Portfolio policies, see our Editorial Policies and the Editorial Policy Checklist.

Statistics

For all statistical analyses, confirm that the following items are present in the figure legend, table legend, main text, or Methods section.

n/a	Confirmed	
☐	☒	The exact sample size (n) for each experimental group/condition, given as a discrete number and unit of measurement
☐	☒	A statement on whether measurements were taken from distinct samples or whether the same sample was measured repeatedly
☐	☒	The statistical test(s) used AND whether they are one- or two-sided<br>*Only common tests should be described solely by name; describe more complex techniques in the Methods section.*
☐	☒	A description of all covariates tested
☐	☒	A description of any assumptions or corrections, such as tests of normality and adjustment for multiple comparisons
☐	☒	A full description of the statistical parameters including central tendency (e.g. means) or other basic estimates (e.g. regression coefficient) AND variation (e.g. standard deviation) or associated estimates of uncertainty (e.g. confidence intervals)
☐	☒	For null hypothesis testing, the test statistic (e.g. F, t, r) with confidence intervals, effect sizes, degrees of freedom and P value noted<br>*Give P values as exact values whenever suitable.*
☒	☐	For Bayesian analysis, information on the choice of priors and Markov chain Monte Carlo settings
☒	☐	For hierarchical and complex designs, identification of the appropriate level for tests and full reporting of outcomes
☐	☒	Estimates of effect sizes (e.g. Cohen's d, Pearson's r), indicating how they were calculated

Our web collection on statistics for biologists contains articles on many of the points above.

Software and code

Policy information about availability of computer code

Data collection	Olympus SZX10 microscope with Improvision Openlab v5.5.2 imaging software; Cell Voyager CV1000 (Yokogawa) with CV1000 Software v1.06.06; Olympus CKX41 with DP21 imaging module; LAS-4000 (Fujifilm) with Multi Gauge software; Zeiss LSM 800 with ZEN2.6 (blue edition) software
Data analysis	MaxQuant version 1.3.0.5; Matlab v2017; GraphPad Prism 8; ImageJ v1.53j; DAVID v6.8

For manuscripts utilizing custom algorithms or software that are central to the research but not yet described in published literature, software must be made available to editors and reviewers. We strongly encourage code deposition in a community repository (e.g. GitHub). See the Nature Portfolio guidelines for submitting code & software for further information.

Data

Policy information about availability of data

All manuscripts must include a data availability statement. This statement should provide the following information, where applicable:

- Accession codes, unique identifiers, or web links for publicly available datasets
- A description of any restrictions on data availability
- For clinical datasets or third party data, please ensure that the statement adheres to our policy

All data for this work are available. Microarray data have been deposited in the NCBI Gene Expression Omnibus (GEO) (Ref 81) with the dataset identifier GSE197631 [https://www.ncbi.nlm.nih.gov/geo/query/acc.cgi?acc=GSE197631] (Gene expression changes in NCU10K cells lacking Nedd4). The mass spectrometry proteomics data have been deposited to the ProteomeXchange via PRIDE (ref 82) partner repository with the dataset identifier PXD024103 [https://www.ebi.ac.uk/pride/archive/projects/PXD024103] (SILAC MS in NCU10K cells lacking Nedd4). Source data are provided with this paper.

Field-specific reporting

Please select the one below that is the best fit for your research. If you are not sure, read the appropriate sections before making your selection.

- [x] Life sciences
- [ ] Behavioural & social sciences
- [ ] Ecological, evolutionary & environmental sciences

For a reference copy of the document with all sections, see nature.com/documents/nr-reporting-summary-flat.pdf

Life sciences study design

All studies must disclose on these points even when the disclosure is negative.

Sample size

N of 3 or more was chosen for all experiments. Power calculations were performed based on variation and effect size from a pilot experiment of cell migration performed with CrispR Nedd4 KO and WT cells.

Data exclusions

No data exclusions included in the manuscript.

Replication

All experiments were performed in triplicate unless stated in the figure legend. Details on specific replication for each experiment are provided in the figure legends.

Randomization

Randomization was performed where possible, such as the passage of the cells used for in vitro experiments, the positioning of WT and Nedd4-/- cells in multiwell plates, positioning of plates in incubators, imaging order of control and Wnt1Cre; Nedd4fl/fl embryos, positioning of explants in multiwells, positioning of transwells in multiwell plates, and western blot apparatus used. Randomization of samples was not possible for experiments in which only 2 testing variables were being analysed.

Blinding

Experiments that were performed blinded include: migration analyses in Fig. 6b, actin repolymerisation in Fig. 7c, ubiquitination assays in Fig. 8a-b and actin repolymerisation in Fig. 8d. These experiments were performed by one investigator and analysed by others that did not know the treatment type or genotype prior to data analysis and measurements being taken. All other experiments were not blinded as they required genotyping and separation of treatment groups which was performed by a single investigator and later validated by others.

Reporting for specific materials, systems and methods

We require information from authors about some types of materials, experimental systems and methods used in many studies. Here, indicate whether each material, system or method listed is relevant to your study. If you are not sure if a list item applies to your research, read the appropriate section before selecting a response.

Materials & experimental systems

n/a	Involved in the study	
[ ]	[x]	Antibodies
[ ]	[x]	Eukaryotic cell lines
[x]	[ ]	Palaeontology and archaeology
[ ]	[x]	Animals and other organisms
[x]	[ ]	Human research participants
[x]	[ ]	Clinical data
[x]	[ ]	Dual use research of concern

Methods

n/a	Involved in the study	
[x]	[ ]	ChIP-seq
[x]	[ ]	Flow cytometry
[x]	[ ]	MRI-based neuroimaging

Antibodies

Antibodies used

Anti-actin (Pierce, AAN02, Clone 7A8.2.1), anti-Sox10 (Abcam, ab155279), anti-Sox10 (R&D, AF2864), anti-Errb3 (AAT Bioquest, Ab-1289), anti-Fndc3b (Abcam, ab135714), anti-Zeb2 (Novus Biologicals, NBP1-82991), anti-Nedd4 (BD, 611481, Lot7297573), anti-ubiquitin P4G7 (Biolegend, 838703), anti-ubiquitin K63 (Cell Signalling Technologies, 5621, Lot3), anti-ubiquitin K48 (Cell Signalling Technologies, 8081, Lot2), anti Pfn1 (Cell Signalling Technologies, 3237, Lot1), anti-RunX2 (Cell Signalling Technologies, 12556, Lot2), anti-CD31 (Biolegend, 102502, clone MEC13.3), anti 14-3-3zeta C-16 (Santa Cruz, sc1019), mouse gamma globulin (Jackson Laboratories, 015-000-002, Lot77861), mouse anti-Flag (Merck, F1804).

Validation

Validation of antibodies has been performed by the manufacturers and in additional publications at the following sites:
Actin AAN02 - https://www.cytoskeleton.com/pdf-storage/datasheets/aan02.pdf.
Sox10 Ab155279: https://www.abcam.com/sox10-antibody-epr4007-ab155279.html.
Sox10 AF2864 - https://resources.rndsystems.com/pdfs/datasheets/af2864.pdf.
v=20220228&_ga=2.80809687.1183973056.1646099672-1410173664.1638832213.
Erbb3 Ab-1289 - https://www.aatbio.com/products/her3-ab-1289-antibody.
Fndc3b - Has been verified to work for western blot (Jinwang Wei, Yuanyuan Sheng, Jianhua Li, Xiaomei Gao, Ning Ren, Qiongzhu Dong, Lunxiu Qin. Genome-Wide Association Study Identifies a Genetic Prediction Model for Postoperative Survival in Patients with Hepatocellular Carcinoma. Med Sci Monit. 2019 Apr 4;25:2452-2478. doi: 10.12659/MSM.915511) and found here to recognise a predominant band in mouse cells at the predicted size in the current study.

	Zeb2 NBP1-82991- https://www.novusbio.com/products/zeb2-antibody_nbp1-82991.<br>Nedd4 611481 - https://www.bdbiosciences.com/en-us/products/reagents/microscopy-imaging-reagents/immunofluorescence-reagents/purified-mouse-anti-mouse-nedd4.611481.<br>Ub P4G7 - https://www.biolegend.com/en-us/products/purified-anti-ubiquitin-antibody-13719.<br>Ub K63 - https://www.cellsignal.com/products/primary-antibodies/k63-linkage-specific-polyubiquitin-d7a11-rabbit-mab/5621.<br>Ub K48 - https://www.cellsignal.com/products/primary-antibodies/k48-linkage-specific-polyubiquitin-d9d5-rabbit-mab/8081?_=1646114806201&Ntt=8081&tahead=true.<br>Pfn1 3237- https://www.cellsignal.com/products/primary-antibodies/profilin-1-antibody/3237.<br>RunX2 12556- https://www.cellsignal.com/products/primary-antibodies/runx2-d1l7f-rabbit-mab/12556?site-search-type=Products&N=4294956287&Ntt=12556%2C&fromPage=plp&_requestid=1708232.<br>CD31 102502- https://www.biolegend.com/en-us/products/purified-anti-mouse-cd31-antibody-380?pdf=true&displayInline=true&leftRightMargin=15&topBottomMargin=15&filename=Purified%20anti-mouse%20CD31%20Antibody.pdf.<br>14-3-3zeta C-16 - specificity to 14-3-3zeta was validated in (H. Ramshaw, X. Xu, E. Jaehne, P. McCarthy, Z. Greenberg, E. Saleh, B. McClure, J. Woodcock, S. Kabbara, S. Wiszniak, T. Wang, C. Parish, M. van den Buuse, B. Baune, A. Lopez & Q. Schwarz. Locomotor hyperactivity in 14-3-3ζ KO mice is associated with dopamine transporter dysfunction. Translational Psychiatry, Dec 3;3:e327 (2013).).<br>Flag - https://www.sigmaaldrich.com/deepweb/assets/sigmaaldrich/product/documents/415/240/f3165dat-mk.pdf.

Eukaryotic cell lines

Policy information about cell lines

Cell line source(s)	The NCU10K neural crest cell line was a gift from Professor Perry Bartlett (Ref 7).<br>293-T cells were originally sourced from ATCC.
Authentication	Cell lines were not authenticated. NCU10K cells were confirmed to express Sox10.
Mycoplasma contamination	No testing for mycoplasma was performed.
Commonly misidentified lines<br>(See ICLAC register)	None.

Animals and other organisms

Policy information about studies involving animals; ARRIVE guidelines recommended for reporting animal research

Laboratory animals	Wnt1-Cre; Nedd4fl/fl; Z/EG animals were used in this study. All mice were maintained on a mixed Sv129 - C57BL/6 background on a 12 hr dark / light cycle at 20-22 C with 40-60% humidity and fed a normal chow diet.
Wild animals	This did not involve wild animals.
Field-collected samples	This did not require field-collected samples.
Ethics oversight	Ethics was obtained from SA Pathology and University of South Australia Animal Ethics Committees, reference number U15/18.

Note that full information on the approval of the study protocol must also be provided in the manuscript.
